# Impact of coronavirus disease on the incidence rate of methicillin-resistant *Staphylococcus aureus* among hospitalized patients with lung cancer: a nationwide retrospective cohort study in Japan

**DOI:** 10.1186/s40780-025-00500-y

**Published:** 2025-10-27

**Authors:** Yasutaka Ihara, Hisafumi Kihara, Waki Imoto, Naoto Okada, Hiroshi Kakeya, Yukihiro Kaneko

**Affiliations:** 1 Clinical Research Promotion Unit, Clinical Therapeutic Trial Center, Ehime University Hospital, 454, Shitsukawa, Toon, Ehime 791-0295 Japan; 2 Center for Data Science, Ehime University, 3, Bunkyo-machi, Matsuyama, Ehime 790-8577 Japan; 3 Integrated Medical and Agricultural School of Public Health, Ehime University, 454, Shitsukawa, Toon, Ehime 791-0295 Japan; 4 Department of Epidemiology and Public Health, Ehime University Graduate School of Medicine, 454, Shitsukawa, Toon, Ehime 791- 0295 Japan; 5 Department of Infection Control Science, Osaka Metropolitan University Graduate School of Medicine, 1-4-3, Asahi-machi, Abeno-ku, Osaka, 545-8585 Japan; 6 Department of Infectious Disease Medicine, Osaka Metropolitan University Hospital, 1-5-7 Asahi-machi, Abeno-ku, Osaka, 545-8586 Japan; 7 Department of Infection Control and Prevention, Osaka Metropolitan University Hospital, 1-5-7 Asahi-machi, Abeno-ku, Osaka, 545-8586 Japan; 8 Research Center for Infectious Disease Sciences (RCIDS), Osaka Metropolitan University Graduate School of Medicine, 1-4-3, Asahi- machi, Abeno-ku, Osaka, 545-8585 Japan; 9 Osaka International Research Center for Infectious Diseases (OIRCID), Osaka Metropolitan University, 1-2-7-601, Asahi-machi, Abeno-ku, Osaka, 545-0051 Japan; 10 Pharmacy Department, Yamaguchi University Hospital, 1-1-1, Minamikogushi, Ube, Yamaguchi, 755-8505 Japan; 11 Department of Bacteriology, Osaka Metropolitan University Graduate School of Medicine, 1-4-3, Asahi-machi, Abeno-ku, Osaka, 545-8585 Japan

**Keywords:** Methicillin-resistant *Staphylococcus aureus*, Coronavirus disease pandemic, Lung cancer, Interrupted time-series analysis

## Abstract

**Background:**

Patients with lung cancer are at increased risk for methicillin-resistant *Staphylococcus aureus* (MRSA) infection, which prolongs treatment and worsens prognosis. Therefore, preventing MRSA infection is critically important in this population. We aimed to investigate whether the incidence rate of MRSA among patients with lung cancer declined after the coronavirus disease (COVID-19) pandemic, owing to the widespread reinforcement of hand-rub use.

**Methods:**

We conducted a nationwide, retrospective, interrupted time-series analysis using a claims database in Japan Medical Data Vision. Hospitalized patients diagnosed with lung cancer between December 2016 and August 2022 were followed for 6 months. MRSA was identified using International Classification of Diseases, 10th Revision, codes in combination with a same-month prescription for an anti-MRSA agent. The incidence rate of MRSA among patients with lung cancer was compared between the pre-COVID (December 2016–April 2020) and post-COVID (April 2020–August 2022) periods using segmented Poisson regression with Newey–West errors and seasonal adjustment.

**Results:**

Among 93,508 eligible patients, 364 developed MRSA. The pre-COVID slope for the incidence rate of MRSA among patients with lung cancer was flat (0.20 per 1,000 person-years/year), whereas the post-COVID slope declined to -8.97 per 1,000 person-years/year. The slope difference (-9.17 per 1,000 person-years/year) indicates a sustained decline in the incidence rate of MRSA among this population after April 2020.

**Conclusions:**

The incidence rate of MRSA among hospitalized patients with lung cancer decreased steadily after the COVID-19 pandemic. These findings suggest that routine hospital-wide infection control measures implemented during the pandemic may yield lasting benefits even in the absence of targeted interventions.

**Supplementary Information:**

The online version contains supplementary material available at 10.1186/s40780-025-00500-y.

## Introduction

Methicillin-resistant *Staphylococcus aureus* (MRSA) remains one of the most prevalent and lethal healthcare-associated pathogens worldwide, prolonging hospital stays, increasing costs, and substantially raising inpatient mortality [1, 2]. Immunocompromised populations, including patients with solid tumors, are especially at risk because cancer-related immune dysregulation, intensive antimicrobial exposure, and frequent use of invasive devices facilitate MRSA colonization and subsequent infection [3].

Lung cancer accounted for an estimated 2.5 million new cases and 1.8 million deaths, ranking second in incidence but first in cancer mortality worldwide [4, 5]. Most individuals with lung cancer undergo repeated hospitalizations for diagnosis, systemic therapy, or management of respiratory complications, circumstances that heighten exposure to nosocomial pathogens such as MRSA [3, 6, 7]. Furthermore, previous research suggests that MRSA infection may enhance metastatic potential in non–small cell lung cancer cells via Toll-like receptor 4/myeloid differentiation factor 88 signaling, implying a potential vicious cycle between MRSA infection and poor prognosis in lung cancer [8]. MRSA control in patients with lung cancer is therefore not only an infection prevention priority but also integral to improving overall outcomes.

Appropriate use of handrub reduces infections caused by resistant organisms, including MRSA [9–12]. The coronavirus disease (COVID-19) pandemic was associated with increased hand-rub use [13, 14]. Importantly, higher hand-rub use was observed in clinics regardless of COVID-19 caseloads, likely reflecting greater awareness of self-protection and infection control measures among healthcare workers and patients. Thus, infection prevention strategies adopted during the COVID-19 pandemic may have indirectly reduced MRSA incidence among patients with lung cancer.

Accordingly, in this study, we sought to determine whether the incidence rate of MRSA among hospitalized patients with lung cancer decreased after the COVID-19 pandemic, using an interrupted time-series analysis (ITSA) design with administrative claims data from Japan.

## Materials and methods

### Data source and study population

This observational study used anonymized administrative claims data supplied by Medical Data Vision (MDV) Co., Ltd. (Tokyo, Japan). The MDV database aggregates diagnosis procedure combinations and receipt records from more than 480 acute-care hospitals and currently contains healthcare encounter data for over 45 million individuals [15]. Several recent clinical studies on infectious diseases have been conducted using the MDV database [16–23]. The available variables include basic demographic characteristics, International Classification of Diseases, 10th Revision (ICD-10) diagnoses, inpatient and outpatient prescriptions, procedural details, dates of service, and survival status.

To identify hospitalized patients newly diagnosed with lung cancer (ICD-10 code C34), we excluded individuals who met the following criteria: (1) a suspected or confirmed diagnosis of MRSA or bacteremia after lung cancer within the last 91.5 days (excluding the index date); and (2) patients managed as outpatients for lung cancer on the index date. A detailed definition of the study population is provided in Supplementary Method 1–2.

### Definition of MRSA

The primary endpoint of this study was the incidence rate of MRSA during hospitalization among patients with lung cancer. This outcome was identified using an algorithm, referring to previous studies [21–23].

First, we identified patients who received a confirmed diagnosis of MRSA (ICD-10 codes and disease codes are shown in Supplementary Table 1) within 6 months of lung cancer diagnosis. Next, to improve specificity, we verified that an anti-MRSA agent (vancomycin hydrochloride, teicoplanin, arbekacin sulfate, linezolid, tedizolid phosphate, or daptomycin) was prescribed in the same month for each patient with a confirmed MRSA diagnosis. This combination of diagnostic and treatment criteria was applied as the definition of MRSA in all primary analyses. For hospitalized patients diagnosed with lung cancer in August 2022, the incidence rate of MRSA among patients with lung cancer was evaluated using databases available in January 2023. To account for MRSA events related to the index hospitalization, any readmission within 30 days of discharge was concatenated to the index hospitalization and treated as a continuous hospital stay.

### Observation period settings

To assess infection prevention measures in healthcare facilities during the COVID-19 pandemic, the study period was divided into two distinct intervals, and the incidence rate of MRSA among hospitalized patients with lung cancer was compared between them. The first interval represented the pre-COVID-19 pandemic period in Japan (December 2016 to April 2020), whereas the second interval represented the post-COVID-19 pandemic period (April 2020 to August 2022).

### Statistical analyses

We conducted an ITSA using a segmented Poisson regression model to evaluate the impact of the COVID-19 pandemic on changes in the slope for the incidence rate of MRSA among hospitalized patients with lung cancer. The Poisson regression model incorporated the log of person-time as the offset. Only the linear term for gradual slope changes was included, while terms for immediate-level changes after the onset of the COVID-19 pandemic were excluded.

The segmented Poisson regression model was specified as $\:log\left({Y}_{t}\right)={\beta\:}_{0}+{\beta\:}_{1}{T}_{t}+{\beta\:}_{2}{X}_{t}{T}_{t}+log\left({pt}_{t}\right)$, where $\:{Y}_{t}$ is the number of incident MRSA cases among hospitalized patients with lung cancer during the 6-month follow-up period for cohort 𝑡 (lung cancer diagnosis month 𝑡); $\:{T}_{t}$ is the time since the start of the study; $\:{pt}_{t}$ is the person-time at risk during the 6-month follow-up for cohort 𝑡; the value of $\:{X}_{t}$ is assigned as 1 for the post-COVID-19 period and 0 for the pre-COVID-19 period; and $\:{X}_{t}{T}_{t}$is the interaction term. Furthermore, $\:{\beta\:}_{0}$ is the intercept and represents the outcome variables at the start of the study and $\:{\beta\:}_{1}$ is the slope in the pre-COVID-19 period. $\:{\beta\:}_{1}+\:{\beta\:}_{2}$ is the slope in the post-COVID-19 period. We also estimated the change in slope between the pre- and post-COVID-19 periods ($\:{\beta\:}_{2})$, which is the parameter of interest.

We conducted the following additional analyses: (1) evaluating the change in the incidence rate of MRSA among hospitalized patients with lung cancer between the pre- and post-COVID-19 periods, stratified by hospital size (< 500 or ≥ 500 beds); (2) replacing the primary outcome with the incidence rate of MRSA bacteremia, defined by the diagnostic codes for MRSA bacteremia in Supplementary Table 1 together with prescription of an anti-MRSA agent; (3) replacing the primary outcome with the incidence rate of blood culture testing within 6 months of lung cancer diagnosis among hospitalized patients; and (4) restricting the cohort to patients who received chemotherapy (Anatomical Therapeutic Chemical code: L01) in the same month as the lung cancer diagnosis.

For all analyses, we used regression models adjusted for seasonality. Results were reported as estimated coefficients with 95% confidence intervals (CI). We applied interrupted time-series models using ordinary least squares regression, incorporating Newey–West standard errors to ensure robustness against heteroscedasticity and autocorrelation. The lag length was set to 3, following a commonly applied rule of thumb based on series length [24]. All analyses were performed using R version 4.4.2 (R Core Team) [25].

The study was designed according to a structured template for implementing real-world evidence studies (Structured template and reporting tool for real-world [STaRT-RWE]) [26]. Furthermore, this study was conducted in accordance with the reporting of studies conducted using the observational routinely collected health data statement for pharmacoepidemiology (RECORD-PE) [27].

## Results

**Fig. 1:**
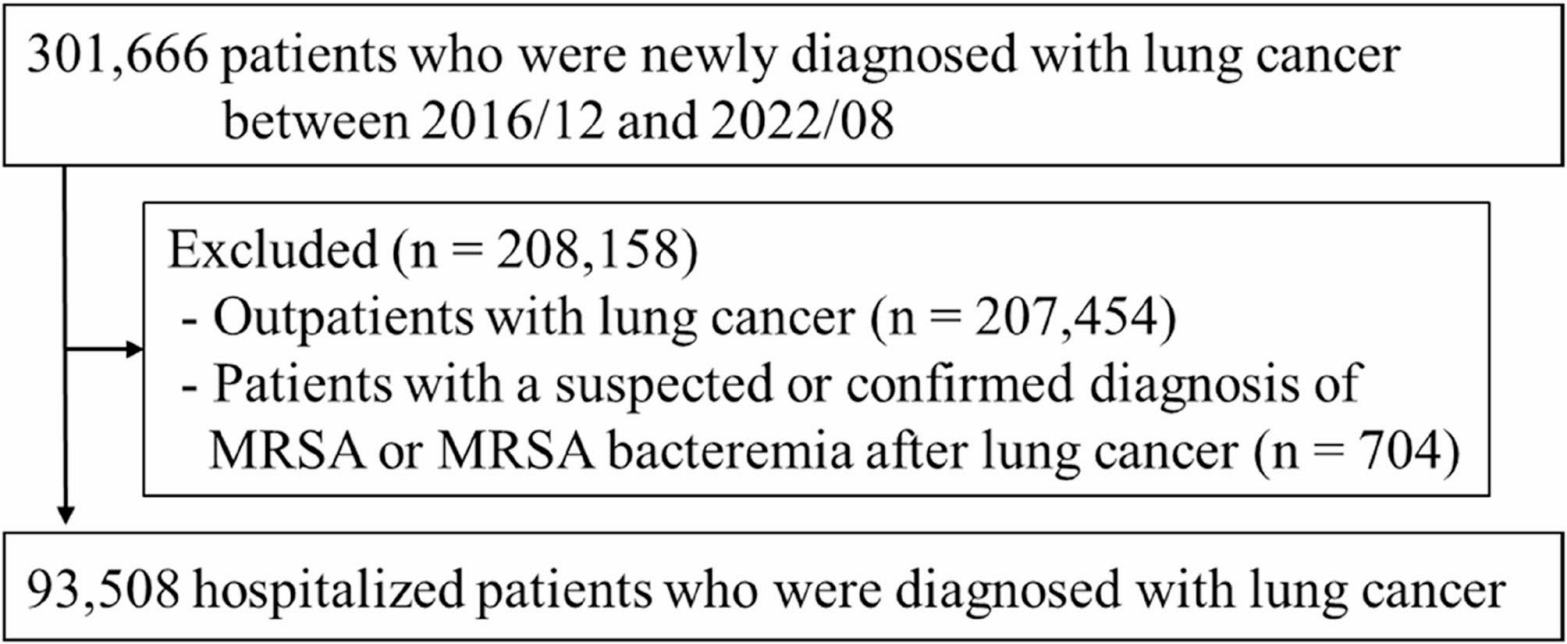
Flowchart of the study population. Abbreviations: MRSA, methicillin-resistant* Staphylococcus aureus*

We identified 93,508 hospitalized patients diagnosed with lung cancer between December 2016 and August 2022 (Fig. 1). Of these, 364 developed MRSA within 6 months of lung cancer diagnosis. Supplementary Fig. 1 presents the number of hospitalized patients newly diagnosed with lung cancer per month. Supplementary Fig. 2 shows the monthly mean length of hospital stay after lung cancer diagnosis. The monthly mean length of hospital stay after lung cancer diagnosis remained stable before and after the COVID-19 pandemic.

**Fig. 2:**
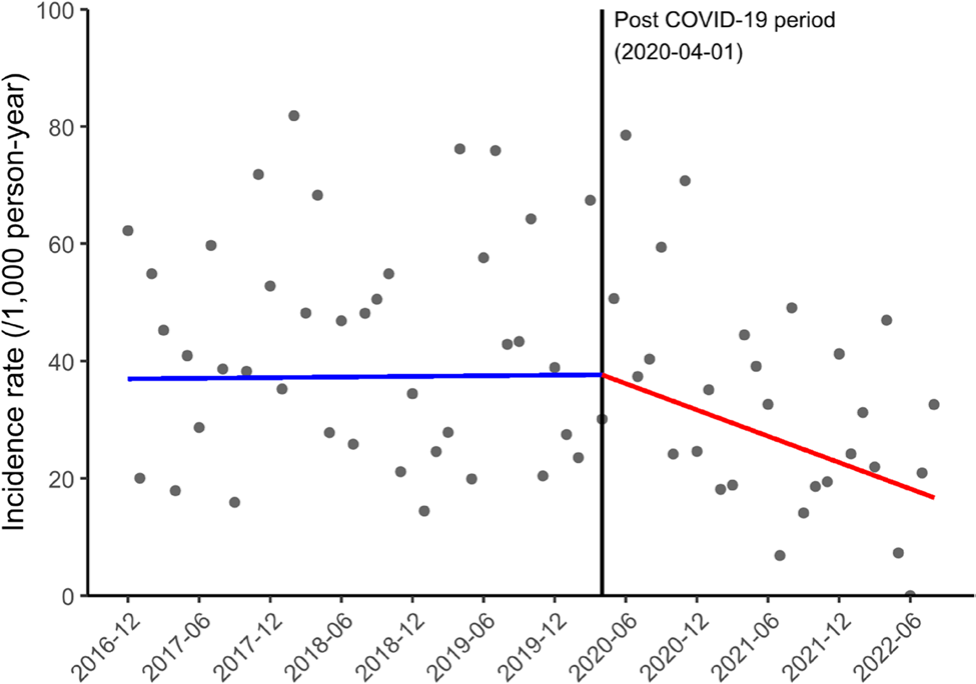
Incidence rate of MRSA among hospitalized patients with lung cancer (per month). Abbreviations: MRSA, methicillin-resistant*Staphylococcus aureus*;COVID-19, coronavirus disease

Figure 2 illustrates changes in the incidence rate of MRSA among hospitalized patients with lung cancer between the pre- and post-COVID-19 periods. The slope for the incidence rate of MRSA decreased from 0.20 per 1,000 person-years/year (95% CI −4.10 to 4.50; *P* = 0.927) to −8.97 per 1,000 person-years/year (95% CI −13.74 to −4.20; *P* < 0.001) in the pre- and post-COVID-19 periods, respectively, and this slope difference of −9.17 per 1,000 person-years/year (95% CI −17.41 to −0.93; *P* = 0.029) was statistically significant (Table 1).

**Table 1:** The incidence rate of MRSA among hospitalized patients with lung cancer

Parameter (per 1,000 person-years/year)	Coefficient [95% CI]	*P* value
Main analysis: Diagnosis and anti-MRSA agent use		
Pre-COVID-19 period slope	0.20 [−4.10 to 4.50]	0.927
Post-COVID-19 period slope	−8.97 [−13.74 to −4.20]	<0.001
Slope difference	−9.17 [−17.41 to −0.93]	0.029
Small/medium hospitals (<500 beds)		
Pre-COVID-19 period slope	−3.22 [−8.23 to 1.79]	0.207
Post-COVID-19 period slope	−8.55 [−14.18 to −2.92]	0.003
Slope difference	−5.33 [−14.44 to 3.78]	0.251
Large hospitals (≥500 beds)		
Pre-COVID-19 period slope	3.61 [−2.35 to 9.57]	0.235
Post-COVID-19 period slope	−9.37 [−15.78 to −2.96]	0.004
Slope difference	−12.98 [−24.87 to −1.09]	0.032

*Abbreviations*: *MRSA* methicillin-resistant *Staphylococcus aureus*,* COVID-19* coronavirus disease, *CI* confidence interval

**Fig. 3:**
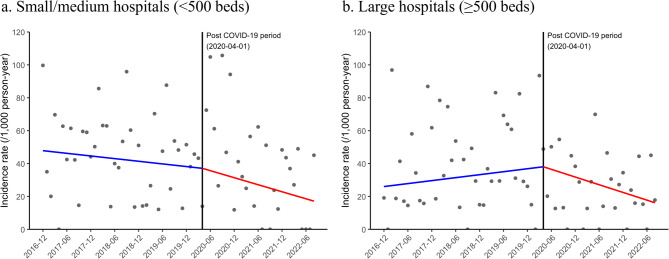
Incidence rate of MRSA among hospitalized patients with lung cancer by hospital size (per month). **a** Small/medium hospitals (<500 beds); (**b**) Large hospitals (≥500 beds). Abbreviations: MRSA, methicillin-resistant *Staphylococcus aureus*; COVID-19, coronavirus disease

Figure 3 shows changes in the incidence rate of MRSA among hospitalized patients with lung cancer between the pre- and post-COVID-19 periods, stratified by hospital size. There were 51,052 patients in small/medium hospitals (< 500 beds) and 42,456 patients in large hospitals (≥ 500 beds). In small/medium and large hospitals, the change in the incidence rate of MRSA between the pre- and post-COVID-19 periods was a decrease of −5.33 per 1,000 person-years/year (*P* = 0.251) and − 12.98 per 1,000 person-years/year (*P* = 0.032), respectively (Table 1). The slope changes between the pre- and post-COVID-19 periods did not differ significantly between large and small/medium hospitals (P for interaction = 0.346).

**Fig. 4:**
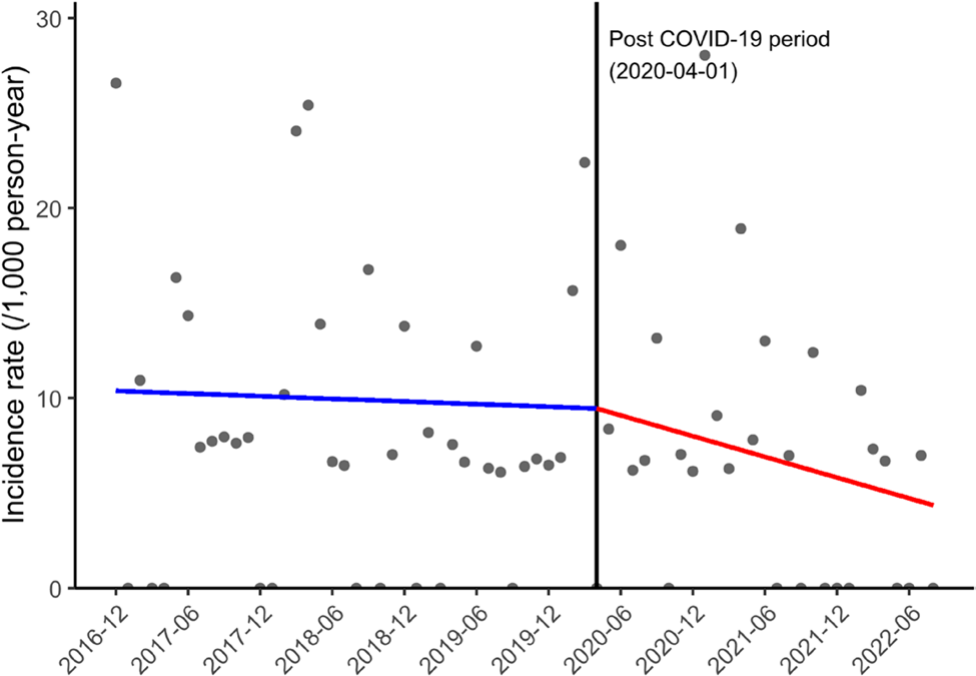
Incidence rate of MRSA bacteremia among hospitalized patients with lung cancer (per month). Abbreviations: MRSA, methicillin-resistant*Staphylococcus aureus*;COVID-19, coronavirus disease

Figure 4 shows changes in the incidence rate of MRSA bacteremia among hospitalized patients with lung cancer between the pre- and post-COVID-19 periods. The slope for the incidence rate of MRSA bacteremia decreased from − 0.28 per 1,000 person-years/year (95% CI −2.87 to 2.32; *P* = 0.834) to −2.18 per 1,000 person-years/year (95% CI −4.50 to 0.14; *P* = 0.066) in the pre- and post-COVID-19 periods respectively, and this slope difference of −1.90 per 1,000 person-years/year (95% CI, −6.43 to 2.63; *P* = 0.411) was not significant (Table 2).

**Table 2:** The incidence rate of MRSA bacteremia among hospitalized patients with lung cancer

Parameter (per 1,000 person-years/year)	Coefficient [95% CI]	*P* value
Pre-COVID-19 period slope	−0.28 [−2.87 to 2.32]	0.834
Post-COVID-19 period slope	−2.18 [−4.50 to 0.14]	0.066
Slope difference	−1.90 [−6.43 to 2.63]	0.411

*Abbreviations*: *MRSA* methicillin-resistant *Staphylococcus aureus*,* COVID-19* coronavirus disease, *CI* confidence interval

Supplementary Fig. 3 presents the results of the main analysis regarding the changes in the incidence rate of blood culture testing among hospitalized patients with lung cancer between the pre- and post-COVID-19 periods. The slope for the incidence rate of blood culture testing among hospitalized patients with lung cancer increased from 29.26 per 1,000 person-years/year (95% CI −8.07 to 66.60; *P* = 0.124) to 83.29 per 1,000 person-years/year (95% CI 40.71 to 125.87; *P* < 0.001) in the pre- and post-COVID-19 periods, respectively; this slope difference of 54.03 per 1,000 person-years/year (95% CI −19.11 to 127.17; *P* = 0.148) was not significant (Supplementary Table 2).

Supplementary Fig. 4 shows the monthly number of hospitalized patients diagnosed with lung cancer and receiving chemotherapy in the same month. The monthly number of hospitalized patients with lung cancer and the number of patients with lung cancer who received chemotherapy remained stable before and after the COVID-19 pandemic. Supplementary Fig. 5 shows changes in the incidence rate of MRSA among hospitalized patients with lung cancer who received chemotherapy between the pre- and post-COVID-19 periods. The slope for the incidence rate of MRSA among patients with lung cancer who received chemotherapy decreased from 3.42 per 1,000 person-years/year (95% CI −10.03 to 16.88; *P* = 0.618) to −16.16 per 1,000 person-years/year (95% CI −29.04 to −3.28; *P* = 0.014) in the pre- and post-COVID-19 periods, respectively, and this slope difference of −19.58 per 1,000 person-years/year (95% CI −44.38 to 5.21; *P* = 0.122) was not significant (Supplementary Table 3).

## Discussion

This study used national data from 93,508 hospitalized patients diagnosed with lung cancer between December 2016 and August 2022 and investigated the incidence rate of MRSA in this population within 6 months after diagnosis. Before the COVID-19 pandemic, the incidence rate of MRSA among patients with lung cancer remained stable, with no increase or decrease. However, after the COVID-19 pandemic, MRSA incidence in this population showed a reduction.

Several studies have evaluated infection control during the COVID-19 pandemic. A 27-hospital Italian study reported increased hand-rub consumption and decreased MRSA from 2020 to 2022 [28]. In Japan, a multicenter report documented an approximately threefold rise in hand-rub use with a concomitant reduction in *S. aureus* bacteremia [29]. Additionally, our previous multicenter investigation showed an immediate increase of approximately 4 mL (from 12.507 to 16.515 mL; *P* < 0.001) in hand-rub use per inpatient following the initiation of COVID-19 admissions [30]. Nationally consistent data from the Japan Nosocomial Infections Surveillance (JANIS) program showed a modest decline in MRSA isolation rates across more than 1,300 hospitals between 2019 and 2020 [31]. Subsequent JANIS surveillance reports indicate that this gradual reduction has been sustained through the most recent reporting period [32]. Taken together, these contemporaneous findings support the plausibility that strengthened hospital-wide infection control, including increased hand-rub use, contributed to the post-COVID decline in MRSA among hospitalized patients with lung cancer. However, because hand-rub compliance could not be directly assessed in the claims dataset analyzed here, this causal interpretation remains assumption-based.

In contrast, during the first wave of the pandemic, a North American hospital reported a temporary surge in blood culture contamination with coagulase-negative staphylococci, a problem attributed to healthcare worker shortages and supply gaps [33]. A multicenter study in the United States found that central venous catheter-related bloodstream infection rates, which had increased early in the pandemic, declined steadily after 2021 once infection-prevention practices were reestablished [34]. Thus, healthcare worker and supply shortages during the early stages of the COVID-19 pandemic may have temporarily elevated healthcare-associated infection rates, and time may have been required to reestablish infection-control measures. This study was conducted over approximately 3 years, starting in April 2020, after the onset of the COVID-19 pandemic. This period was sufficiently long to capture the stabilizing effects of infection-control practices. The long-term follow-up from the onset of the COVID-19 pandemic in the present study demonstrates the value of continuing research even after the initial emergency has subsided. Our findings suggest that, as infection-control routines became established, MRSA incidence among patients with lung cancer decreased annually, indicating sustained benefits. This highlights the importance of maintaining core infection-control practices—such as hand-rub use, meticulous catheter and vascular-access care, and rapid audit and feedback—even when new crises demand attention. When applied consistently, these measures provide sustained protection against MRSA in hospitalized patients with lung cancer.

This study revealed three findings regarding infection control in patients with lung cancer. First, patients with lung cancer are often frail and require frequent hospital visits, putting them at risk of healthcare-associated infections [3, 6, 7]. Together with previous studies on infection-control measures during the COVID-19 pandemic, such as hand-rub use, this study indicates that simple hospital-wide infection-control practices, without special interventions, can protect patients with lung cancer from MRSA. Second, a post-COVID decline in MRSA among patients with lung cancer was observed in both small/medium and large hospitals. As demonstrated by previous studies [28, 35], evidence-based infection-control practices provide equal benefit in small/medium and large hospitals as long as healthcare worker adherence is maintained. Third, the sustained decline in MRSA bacteremia observed in our study may reflect not only reduced transmission via direct and indirect contact [36] but also better maintenance of vascular access and overall adherence to infection-control practices during the COVID-19 pandemic. Although MRSA bacteremia represents a bloodstream infection, it is often preceded by MRSA colonization or local infection, which can be introduced into the bloodstream through invasive procedures or catheter insertion. Thus, hospital-wide improvements in hand-rub use and contact precautions may have reduced MRSA colonization and subsequently decreased catheter-related bloodstream infections. Previous studies have reported that catheter-related MRSA bacteremia can be prevented through consistent hand-rub use, aseptic catheter insertion, and appropriate maintenance of vascular-access devices [37, 38]. In this study, only a slight slope difference in of the incidence rate of MRSA bacteremia was observed between the pre- and post-COVID-19 periods, and the difference was not significant. However, for patients with lung cancer, in whom MRSA bacteremia is associated with high short-term mortality [39], even a modest decline is clinically important.

This study has some limitations. First, there was an issue regarding the outcome evaluation period. Outcomes were evaluated based on the development of MRSA within 6 months of lung cancer diagnosis. Therefore, patients diagnosed immediately before the onset of the COVID-19 pandemic (e.g., March 2020) may have developed MRSA infection during the post-COVID-19 pandemic period, despite being classified in the pre-COVID-19 period. This misclassification of partial exposure may have underestimated the effects of infection-control measures during the COVID-19 pandemic by reducing the pre-COVID slope. Therefore, the observed slope changes are conservative; the true impact of infection-control measures during the COVID-19 pandemic on the incidence rate of MRSA among patients with lung cancer may have been greater than the estimated values. Second, the administrative data lacked microbiological confirmation, which may have led to misclassification of MRSA. However, misclassification is probably nondifferential over time; when the misclassification rate is stable before and after the interruption, it mainly attenuates the overall incidence level and has little influence on slope estimates that drive an ITSA, meaning our inference on the pre- and post-COVID-19 trend should remain robust. Third, MRSA may have been underdiagnosed. The incidence rate of blood culture testing among hospitalized patients with lung cancer increased after the COVID-19 pandemic. Therefore, it is likely that MRSA underdiagnosis was minimal. However, this increase may reflect a higher number of patients with bloodstream infections requiring blood cultures. In addition, many other changes occurred during the pandemic that could not be captured in our data, such as visitor restrictions, reduced hospital admissions, and staffing challenges. These factors may also have influenced MRSA incidence and should be considered potential unmeasured confounders. The COVID-19 pandemic may have contributed to an increase in other infectious diseases, warranting further investigation in future studies. Fourth, because this database does not capture whether hospitals qualify for additional healthcare reimbursement for infection prevention and control, we cannot examine the distribution of facilities included according to their infection-control infrastructure. Consequently, the external validity of our findings may be limited, as the extent to which the observed decline in MRSA incidence applies to hospitals without formal infection control incentives remains uncertain. Fifth, although our cohort included patients who initiated chemotherapy during hospitalization for newly diagnosed lung cancer, indicating that most MRSA events likely represented hospital-acquired infections, we could not explicitly distinguish community-acquired from nosocomial cases using this database. Because community-onset MRSA is less likely to be influenced by hospital-level infection control practices, this limitation should be considered when interpreting our findings. Finally, some hospitalized patients may have concurrently had COVID-19 and therefore received enhanced infection-control measures, including isolation, personal protective equipment, and restricted contact. Although the number of such cases within our lung cancer cohort was likely small, these precautions may have influenced MRSA incidence and should also be considered when interpreting our findings.

## Conclusion

We showed that the incidence rate of MRSA among hospitalized patients with lung cancer decreased after the COVID-19 pandemic. These findings suggest that the implementation of infection control measures during the COVID-19 pandemic, such as increased hand-rub use and general hospital-wide precautions, even without disease-specific interventions, can protect frail patients with lung cancer from MRSA. Therefore, the routine infection control measures adopted during the pandemic may provide long-term benefits.

## Supplementary Information

Supplementary Material 1.

## Data Availability

The data that support the findings of this study are available from Medical Data Vision Co., Ltd. Restrictions apply to the availability of these data, which were used under license for this study. Data are available from the authors with the permission of Medical Data Vision Co., Ltd.

## Abbreviations

MRSA: Methicillin-resistant *Staphylococcus aureus*
COVID-19: Coronavirus disease
ITSA: Interrupted time-series analysis
MDV: Medical Data Vision
ICD-10: International Classification of Diseases 10th Revision
CI: Confidence intervals
STaRT-RWE: Structured template and reporting tool for real world
RECORD-PE: Reporting of studies conducted using observational routinely collected health data statement for pharmacoepidemiology
JANIS: Japan Nosocomial Infections Surveillance
