# Supplementary material for: Impact of coronavirus disease on the incidence rate of methicillin-resistant Staphylococcus aureus among hospitalized patients with lung cancer: a nationwide retrospective cohort study in Japan
Source: J Pharm Health Care Sci. 2025 Oct 27;11:95. doi: 10.1186/s40780-025-00500-y (PMC12560535; doi:10.1186/s40780-025-00500-y)
Supplement: Supplementary file 1 — Supplementary Material 1. [file 40780_2025_500_MOESM1_ESM.docx]

**Impact of coronavirus disease on the incidence rate of methicillin-resistant *Staphylococcus aureus* among hospitalized patients with lung cancer: a nationwide retrospective cohort study in Japan**

Yasutaka Ihara^a,b,c#*^, Hisafumi Kihara^c,d#^, Waki Imoto^e,f,g,h,i^, Naoto Okada^j^, Hiroshi Kakeya^e,f,g,h,i^, Yukihiro Kaneko^h,i,k^

1. ^Clinical Research Promotion Unit, Clinical Therapeutic Trial Center, Ehime University Hospital, Ehime, Japan, 454, Shitsukawa, Toon, Ehime 791-0295, Japan^
2. ^Center for Data Science, Ehime University, 3, Bunkyo-machi, Matsuyama, Ehime, 790-8577, Japan^
3. ^Integrated Medical and Agricultural School of Public Health, Ehime University, 454, Shitsukawa, Toon, Ehime, 791-0295, Japan^
4. ^Department of Epidemiology and Public Health, Ehime University Graduate School of Medicine, 454, Shitsukawa, Toon, Ehime, 791-0295, Japan^
5. ^Department of Infection Control Science, Osaka Metropolitan University Graduate School of Medicine, Osaka, 545-8585, Japan^
6. ^Department of Infectious Disease Medicine, Osaka Metropolitan University Hospital, 1-5-7 Asahi-machi, Abeno-ku, Osaka, 545-8586, Japan^
7. ^Department of Infection Control and Prevention, Osaka Metropolitan University Hospital, 1-5-7 Asahi-machi, Abeno-ku, Osaka, 545-8586, Japan^
8. ^Research Center for Infectious Disease Sciences (RCIDS), Osaka Metropolitan University Graduate School of Medicine, 1-4-3, Asahi-machi, Abeno-ku, Osaka, 545-8585, Japan^
9. ^Osaka International Research Center for Infectious Diseases (OIRCID), Osaka Metropolitan University, 1-2-7-601, Asahi-machi, Abeno-ku, Osaka, 545-0051, Japan^
10. ^Pharmacy Department, Yamaguchi University Hospital, 1-1-1, Minamikogushi, Ube, Yamaguchi, 755-8505, Japan^
11. ^Department of Bacteriology, Osaka Metropolitan University Graduate School of Medicine, 1-4-3, Asahi-machi, Abeno-ku, Osaka, 545-8585, Japan^

^#^ These authors contributed equally to this work.

**Table of contents**

**Supplementary Method 1.** Definitions of patients who were newly diagnosed with lung cancer

**Supplementary Method 2.** Eligibility Criteria

**Supplementary Table 1.** ICD-10 codes and disease codes for MRSA and MRSA bacteremia

**Supplementary Table 2.** The incidence rate of blood culture testing among hospitalized patients with lung cancer

**Supplementary Table 3.** The incidence rate of MRSA among hospitalized patients with lung cancer who received chemotherapy

**Supplementary Figure 1.** The number of hospitalized patients diagnosed with lung cancer (per month)

**Supplementary Figure 2.** Mean length of hospital stays after lung cancer diagnosis (per month)

**Supplementary Figure 3.** Incidence rate of blood culture testing among hospitalized patients with lung cancer (per month)

**Supplementary Figure 4**. The number of hospitalized patients with lung cancer who received chemotherapy (per month)

**Supplementary Figure 5**. Incidence rate of MRSA among hospitalized patients with lung cancer who received chemotherapy (per month)

Abbreviations: ICD-10, International Statistical Classification of Diseases and Related Health Problems, Tenth Revision; MRSA, methicillin-resistant *Staphylococcus aureus*

**Supplementary Method 1.** Definitions of patients who were newly diagnosed with lung cancer

**
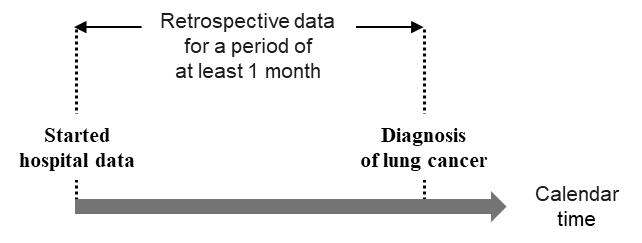
**

To identify patients who were newly diagnosed with lung cancer, we included those with a minimum 1-month interval from database entry to the date of first lung cancer diagnosis. In this study, the month of the lung cancer diagnosis was recorded as the index date.

**Supplementary Method 2.** Eligibility Criteria


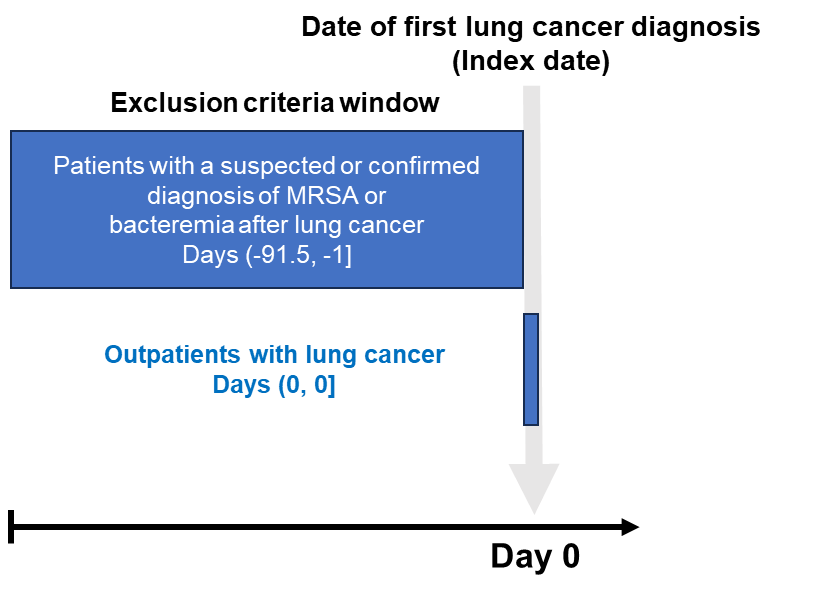


Patients who met any of the following criteria were excluded from the study: (1) patients with a suspected or confirmed diagnosis of MRSA or bacteremia after lung cancer within the last 91.5 days (excluding index date); and (2) outpatients with lung cancer on the index date.

Abbreviations: MRSA, methicillin-resistant *Staphylococcus aureus*

**Supplementary Table 1.** ICD-10 codes and disease codes for MRSA and MRSA bacteremia

| **Disease name** | **Disease code** | **ICD-10 code** | **MRSA**  **(Applicable:〇, Not applicable:×)** | **MRSA bacteremia**  **(Applicable:〇,**  **Not applicable:×)** |
| --- | --- | --- | --- | --- |
| MRSA enteritis | 20083774 | A048 | 〇 | × |
| MRSA bacteremia | 20105870 | A410 | 〇 | 〇 |
| MRSA sepsis | 20083764 | A410 | 〇 | 〇 |
| MRSA infection | 20050274 | A490 | 〇 | × |
| MRSA meningitis | 20083771 | G003 | 〇 | × |
| MRSA conjunctivitis | 20107417 | H109 | 〇 | × |
| MRSA keratoconjunctivitis | 20107419 | H162 | 〇 | × |
| MRSA endophthalmitis | 20107418 | H440 | 〇 | × |
| MRSA otitis media | 20107420 | H669 | 〇 | × |
| MRSA infective endocarditis | 20083765 | I330 | 〇 | × |
| MRSA rhinitis | 20107421 | J00 | 〇 | × |
| MRSA pneumonia | 20083769 | J152 | 〇 | × |
| MRSA pulmonary abscess | 20083768 | J852 | 〇 | × |
| MRSA empyema | 20083772 | J869 | 〇 | × |
| MRSA peritonitis | 20083770 | K650 | 〇 | × |
| MRSA arthritis | 20083767 | M0009 | 〇 | × |
| MRSA shoulder arthritis | 20100882 | M0001 | 〇 | × |
| MRSA hip arthritis | 20097580 | M0005 | 〇 | × |
| MRSA knee arthritis | 20097648 | M0006 | 〇 | × |
| MRSA elbow arthritis | 20099732 | M0002 | 〇 | × |
| MRSA osteomyelitis | 20083766 | M8699 | 〇 | × |
| MRSA cystitis | 20088190 | N308 | 〇 | × |
| MRSA surgical site infection | 20083773 | T814 | 〇 | × |
| Transient bacteremia | 20054436 | A499 | × | 〇 |
| Intermittent bacteremia | 20057188 | A499 | × | 〇 |
| Bacteremia | 20059341 | A499 | × | 〇 |
| Persistent bacteremia | 20063197 | A499 | × | 〇 |

Abbreviations: ICD-10, International Statistical Classification of Diseases and Related Health Problems, Tenth Revision; MRSA, methicillin-resistant *Staphylococcus aureus*

**Supplementary Table 2.** The incidence rate of blood culture testing among hospitalized patients with lung cancer

| Parameter (per 1,000 person-years/year) | | Coefficient [95% CI] | P value |
| --- | --- | --- | --- |
|  | Pre-COVID-19 period Slope | 29.26 [-8.07 to 66.60] | 0.124 |
|  | Post-COVID-19 period Slope | 83.29 [40.71 to 125.87] | <0.001 |
|  | Slope difference | 54.03 [-19.11 to 127.17] | 0.148 |
| Abbreviations: COVID-19, coronavirus disease; CI, confidence interval | | | |

**Supplementary Table 3.** The incidence rate of MRSA among hospitalized patients with lung cancer who received chemotherapy

| Parameter (per 1,000 person-years/year) | | Coefficient [95% CI] | P value |
| --- | --- | --- | --- |
|  | Pre-COVID-19 period Slope | 3.42 [-10.03 to 16.88] | 0.618 |
|  | Post-COVID-19 period Slope | -16.16 [-29.04 to -3.28] | 0.014 |
|  | Slope difference | -19.58 [-44.38 to 5.21] | 0.122 |
| Abbreviations: MRSA, methicillin-resistant *Staphylococcus aureus*; COVID-19, coronavirus disease; CI, confidence interval | | | |

**Supplementary Figure 1.** The number of hospitalized patients diagnosed with lung cancer (per month)

**
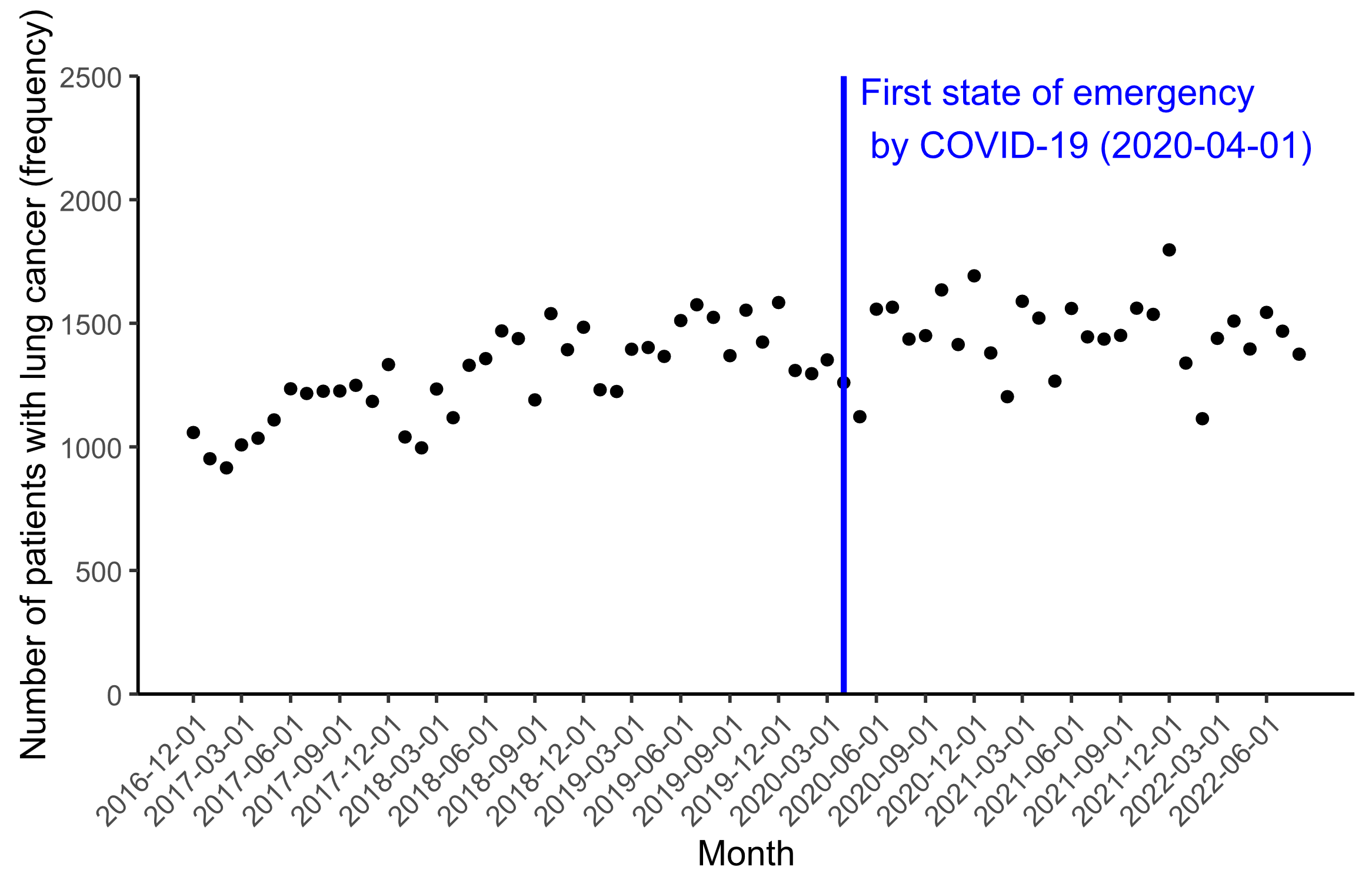
**

Abbreviations: COVID-19, coronavirus disease

**Supplementary Figure 2.** Mean length of hospital stays after lung cancer diagnosis (per month)


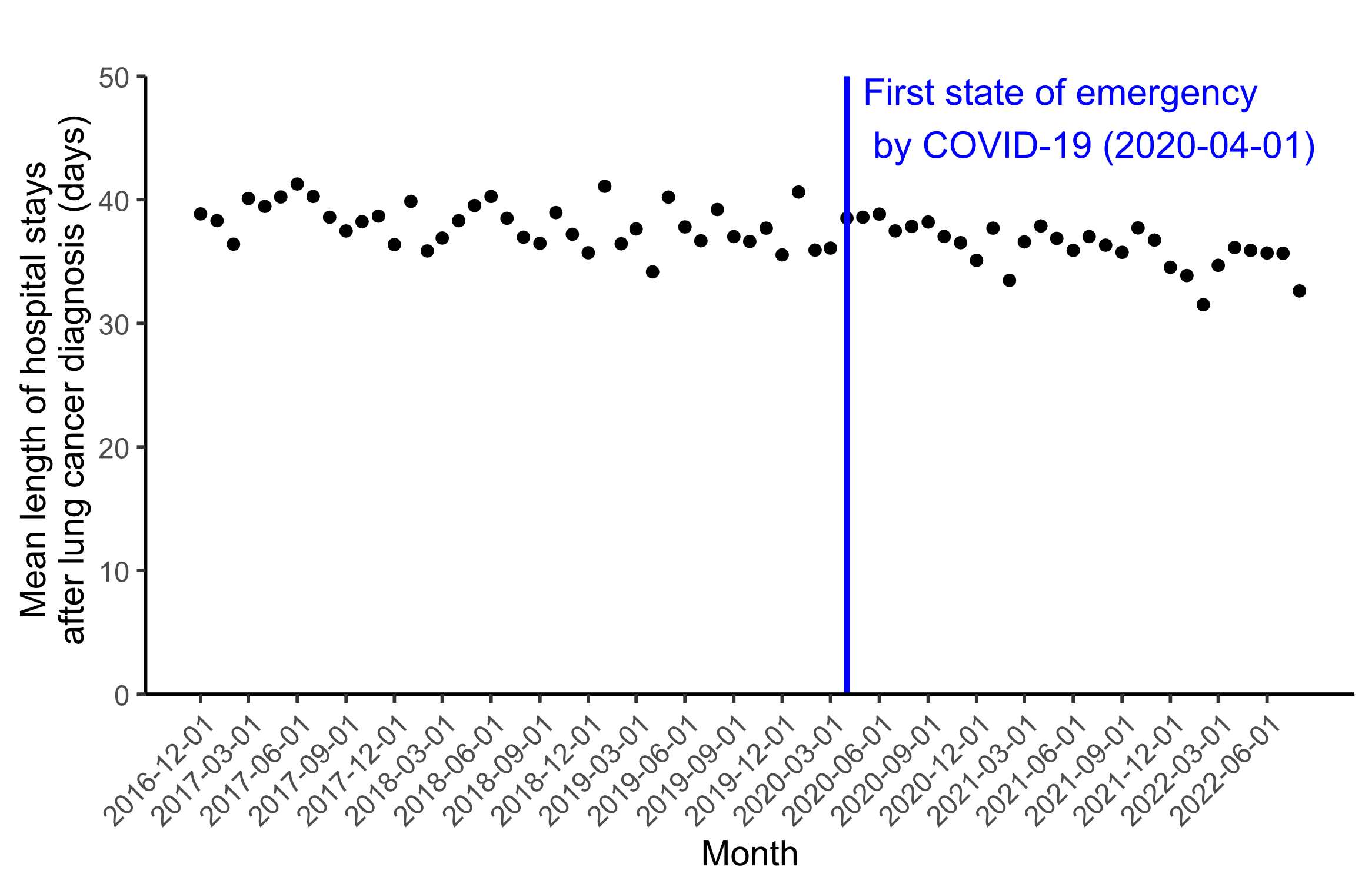


Given that the mean length of hospital stay was comparable before and after the COVID-19 pandemic, the observed decrease in the incidence rate of MRSA is unlikely to be attributable to shorter hospitalization.

Abbreviations: COVID-19, coronavirus disease; MRSA, methicillin-resistant *Staphylococcus aureus*

**Supplementary Figure 3.** Incidence rate of blood culture testing among hospitalized patients with lung cancer (per month)


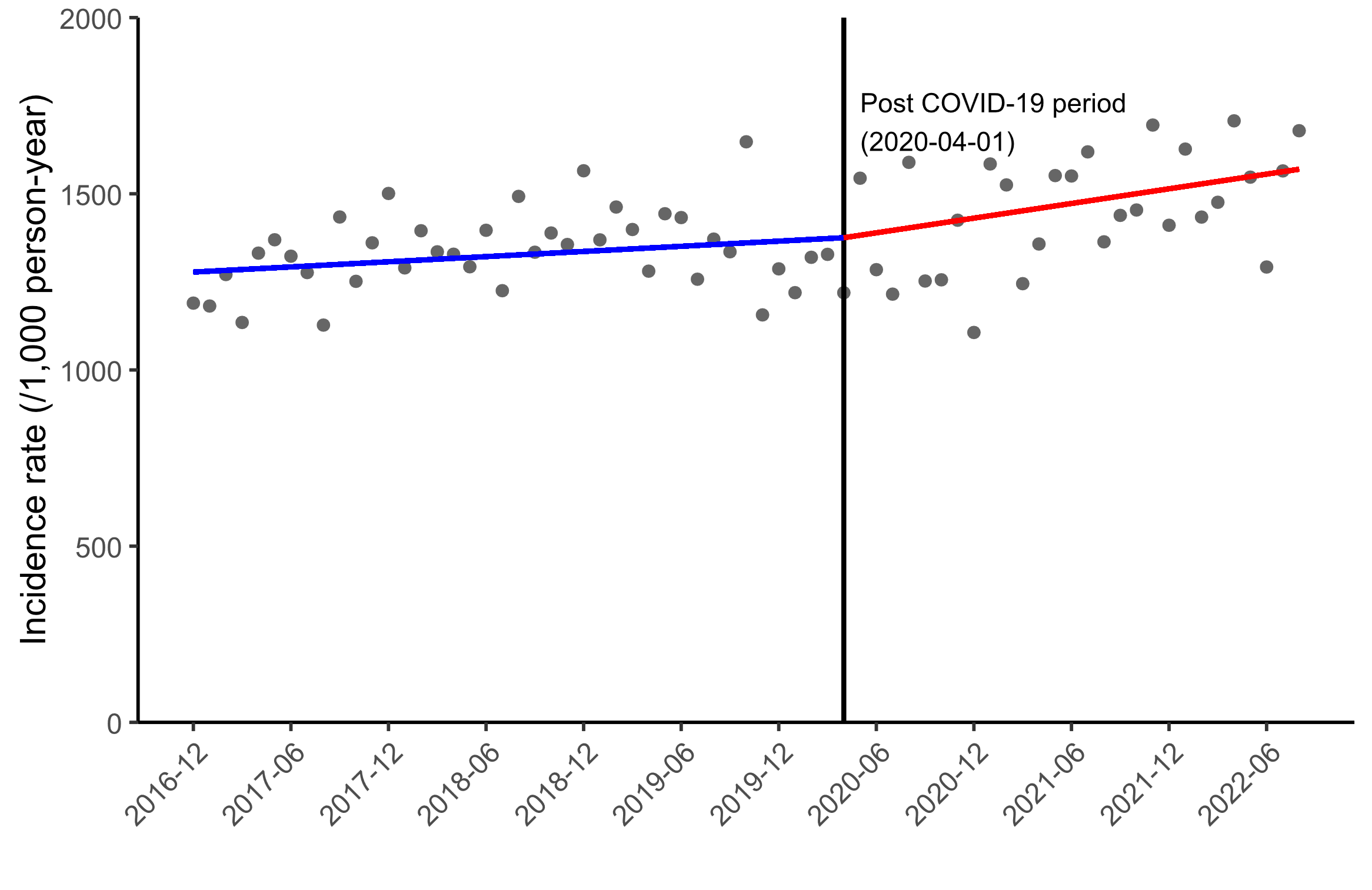


Abbreviations: COVID-19, coronavirus disease

**Supplementary Figure 4.** The number of hospitalized patients with lung cancer who received chemotherapy (per month)


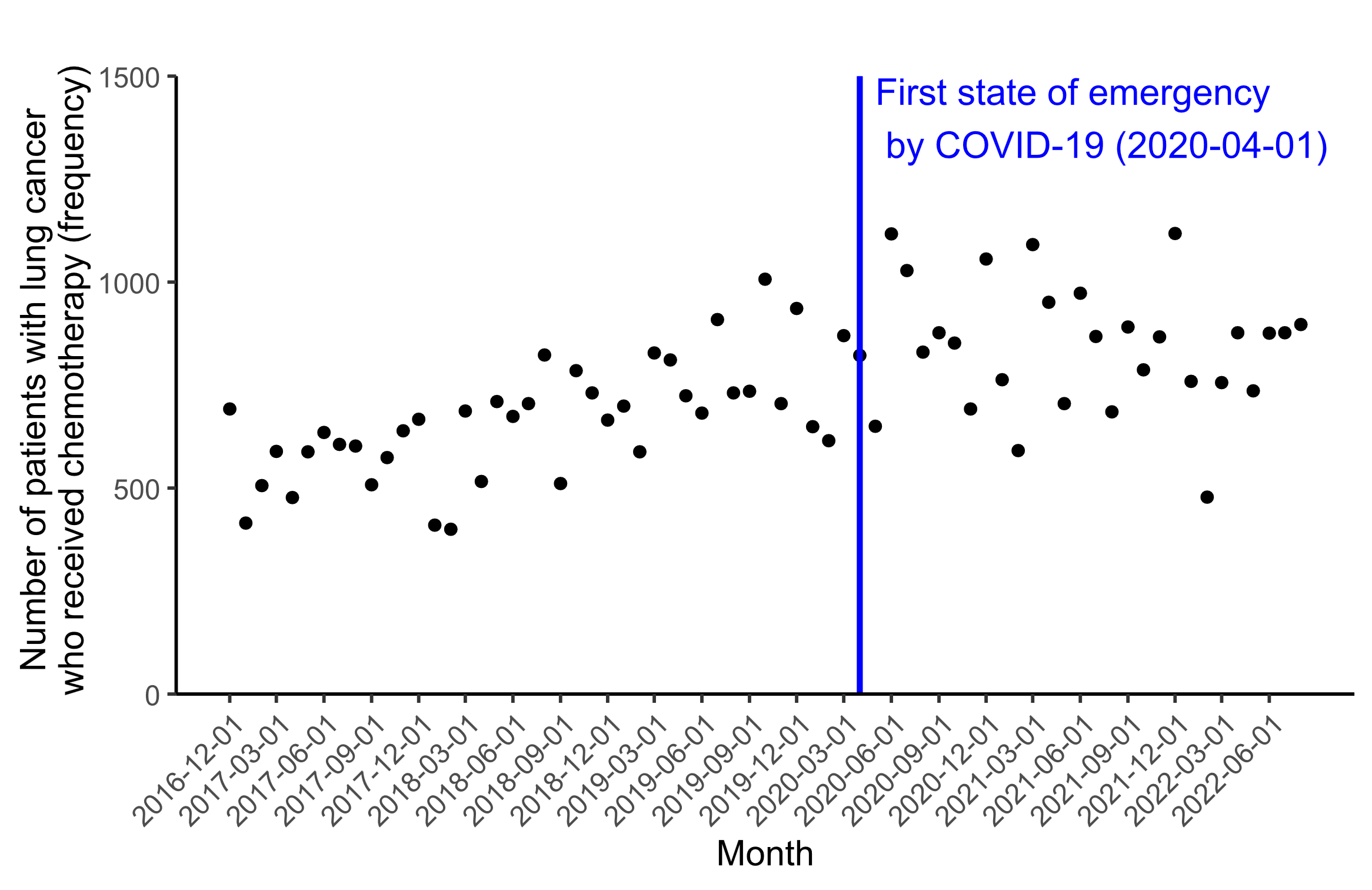


Abbreviations: COVID-19, coronavirus disease

**Supplementary Figure 5**. Incidence rate of MRSA among hospitalized patients with lung cancer who received chemotherapy (per month)


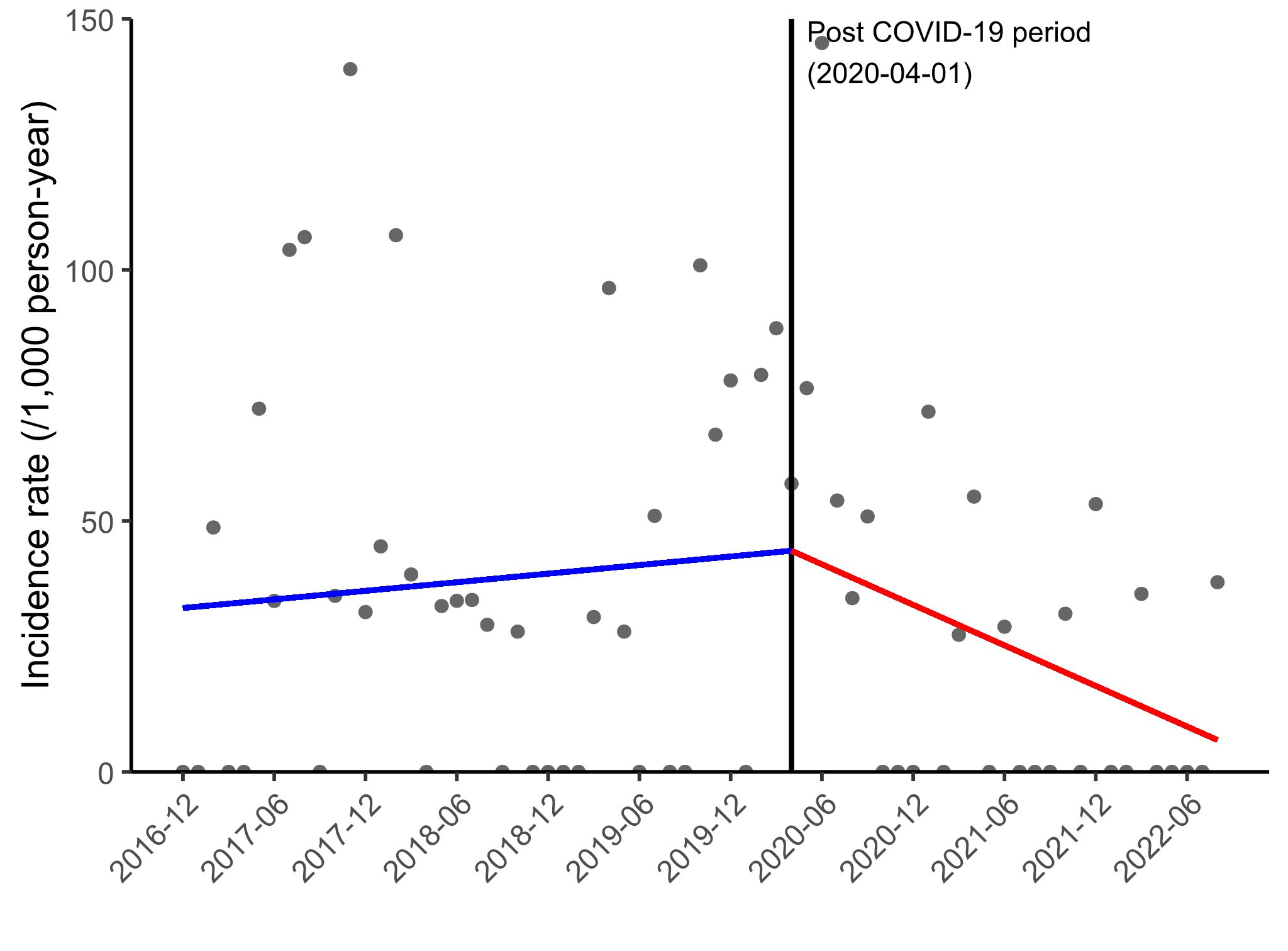


Abbreviations: MRSA, methicillin-resistant *Staphylococcus aureus*; COVID-19, coronavirus disease
